# Novel Divergent Rhabdovirus in Feces of Red Fox, Spain

**DOI:** 10.3201/eid2012.140236

**Published:** 2014-12

**Authors:** Rogier Bodewes, Aritz Ruiz-Gonzalez, Anita C. Schürch, Albert D.M.E. Osterhaus, Saskia L. Smits

**Affiliations:** Erasmus Medical Centre, Rotterdam, the Netherlands (R. Bodewes, A.C. Schürch, A.D.M.E. Osterhaus, S.L. Smits);; University of the Basque Country, Vitoria-Gasteiz, Spain (A. Ruiz-Gonzalez);; National Institute for Environmental Protection and Research, Ozzano dell'Emilia, Italy (A. Ruiz-Gonzalez);; Viroclinics Biosciences, Rotterdam (A.D.M.E. Osterhaus, S.L. Smits)

**Keywords:** rhabdovirus, viral metagenomics, wildlife, red fox, fox, viruses, Spain

**To the Editor:** Rhabdoviruses (family *Rhabdoviridae*) are enveloped single-stranded negative-sense RNA viruses belonging to the Mononegavirale*s* order. The International Committee on Taxonomy of Viruses recognizes 11 genera (*Cytorhabdovirus*, *Ephemerovirus*, *Lyssavirus*, *Novirhabdovirus*, *Nucleorhabdovirus*, *Perhabdovirus*, *Sigmavirus*, *Sprivivirus*, *Tibrovirus*, *Tupavirus*, *Vesiculovirus*) (*1*). In addition, many recently described rhabdoviruses remain unassigned. Rhabdoviruses contain 5 major genes, encoding for nucleoprotein (N), phosphoprotein (P), matrix (M), glycoprotein (G), and RNA-dependent RNA polymerase (L). The *Rhabdoviridae* family includes pathogens of various animal species, humans, and plants. Viruses of the genus *Lyssavirus* are the most relevant to public health because they can cause rabies. Bats are the driving force within this genus; foxes and various other species of wild carnivores also can be infected with lyssaviruses and transmit them to humans and dogs (*2*).

During a viral metagenomic survey, conducted as described previously (*3*), of fecal samples collected from 4 red foxes (*Vulpes vulpes*) that were found dead in Álava, Basque Country, Spain, we identified the complete coding sequence and the partial leader and trailer sequence of a novel rhabdovirus, tentatively called red fox fecal rhabdovirus (RFFRV; 15,541 nt, GenBank accession no. KF823814; Technical Appendix) by mapping 8,287 of the 56,519 sequence reads in the sample of a red fox. A proportion of obtained reads contained sequences that were >99% identical to mitochondrial DNA of *V. vulpes*, which confirmed that the sample was collected from a red fox.

The obtained sequence of RFFRV was partially confirmed by specific primers and Sanger sequencing of PCR amplicons. Five major and 3 minor open reading frames (ORFs) were identified that had a genome organization similar to that of other rhabdoviruses (Figure, panel A). No significant hits were obtained by BLAST analysis (http://blast.ncbi.nlm.gov/Blast.cgi) of N, P, M, and G nucleotide and amino acid sequences, which was reported previously for novel divergent rhabdoviruses (*4*).

**Figure:**
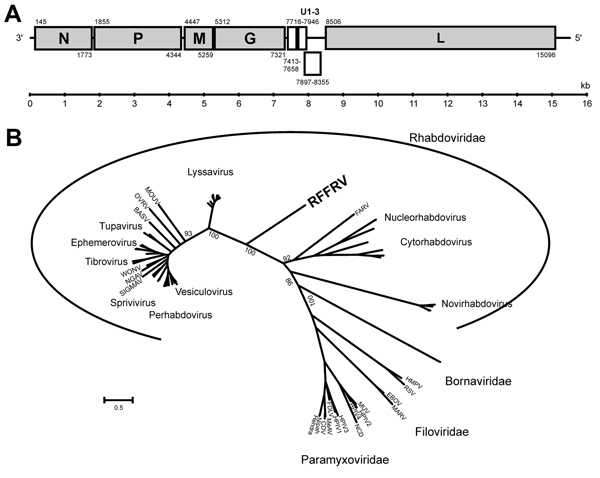
Genome organization and phylogenetic analysis of RFFRV. A) Genome organization of RFFRV. Indicated are the locations of the major ORFs (including the positions of the first and last nucleotide) and 3 theoretical minor ORFs between the G and L genes. B) Phylogenetic maximum-likelihood tree using the WAG+F+I+G model and 100 bootstrap replicates in MEGA5 (http://www.megasoftware.net) of the deduced amino acid sequence of the L genes of various viruses of the order Mononegavirales. G, glycoprotein; L, RNA-dependent RNA polymerase; M, matrix; N, nucleoprotein; ORF, open reading frame; P, phosphoprotein; RFFRV, RFFRV, red fox fecal rhabdovirus. Only bootstrap values in the close proximity of the branch of the RFFRV are indicated. Scale bar indicates nucleotide substitutions per site. Viruses and GenBank accession numbers: vesicular stomatitis virus, Indiana, AAA48441; Maraba virus, AEI52253; cocal virus, EU373657; vesicular stomatitis virus, New Jersey, M20166; Jurona virus, HM566194; Perinet virus, HM566195; Chandipura virus, HM627187; Isfahan virus, AJ810084; pike fry rhabdovirus, ACP28002; spring viremia of carp virus, NC_002803; eel virus European, X JX827265; Siniperca chuatsi rhabdovirus, NC_008514; SIGMAV, sigma virus, Q410979; NGAV, Ngaingan virus, YP003518294; WONV, Wongabel virus, YP002333280; Tupaia virus, NC_007020; Durham virus, FJ952155; Kotonkan virus, NC_017714; Adelaide river virus, JN935380; Obodhiang virus, YP006200965; bovine ephemeral fever virus, NC_002526; Kimberley virus, JQ941664; Tibrogargan virus, GQ294472; Chandipura virus, GQ294473; BASV, Bas-Congo virus, JX297815; OVRV, Oak-Vale virus, AEJ07650; MOUV, moussavirus, FJ985749; Ikoma lyssavirus, JX193798; West Caucasian bat virus, EF614258; Lagos bat virus, JX901139; Shimoni bat virus, ADD84511; Mokola virus, YP142354; Irkut virus, EF614260; lyssavirus ozernoe, FJ905105; European bat lyssavirus 1, NC_009527; Duvenhage virus, JN986749; rabies virus (strain SRV9), AAT48626; Australian bat lyssavirus, NC_003243; Aravan virus, EF614259; Khujand virus, EF614261; FARV, Farmington virus, HM627182; rice yellow stunt virus, NC_003746; taro vein chlorosis virus, NC_006942; northern cereal mosaic virus, NP_597914; strawberry crinkle virus, AAQ97583; lettuce necrotic yellows virus, NC_007642; lettuce yellow mottle virus, YP_002308376; viral hemorrhagic septicemia virus, AB672614; snakehead rhabdovirus, NC 000903; infectious hematopoietic necrosis virus, L40883, Hirame rhabdovirus, NC_005093; bornavirus, L27077; bornavirus, NP_042024; HMPV, human metapneumovirus, AF371337; RSV, human respiratory syncytial virus, NP_056866; EBOV, Zaire ebolavirus, AAG40171; MARV, Marburg marburgvirus, YP_001531159; MUV, mumps virus, NC_002200; HPIV, human parainfluenza virus, CAA40788; HPIV, 4a, BAJ11747; Newcastle disease virus, ADH10207; HPIV, AAA46854; HPIV, AAA69579; FDLV, fer-de-lance paramyxovirus, NP_899661; MeAV, measles virus, NC_001498; CDV, canine distemper virus, AAR32274; Nipah virus, AAY43917; Hendra virus, NP_047113.

Predicted N, P, and M genes of RFFRV consist of 1,629, 2,490, and 813 nt, respectively, encoding for 543, 830, and 271 aa (Technical Appendix Table 1). In addition to the absence of significant hits observed by BLAST analysis, no significant sequence homology was observed with known rhabdovirus proteins in pairwise alignments. Furthermore, no conserved motifs were detected in N, P, and M genes of RFFRV that are commonly observed in rhabdoviruses. However, intergenic regions between all major ORFs contained relatively conserved motifs that could be transcription termination/polyadenylation sequences (A/U) CU_7_, similar to other rhabdoviruses (*5*). Adjacent to this termination signal was a stretch of conserved nucleotides that might function as a transcription initiation signal Technical Appendix Table 1).

The amino acid sequence of the G protein consisted of 669 aa and contained an N terminal signal peptide (1-MYHLIVLLVMLGQRAVA-17), a noncytoplasmic domain (aa 18–646), a transmembrane domain (647-ITAILMPLLSLAVVVGIIMCC-667), and a cytoplasmic tail of 2 aa, similar to other rhabdovirus G proteins as predicted by using Phobius and TMHMM (http://www.cbs.dtu.dk/services/TMHMM) (*6*,*7*). We predicted 3 potential glycosylation sites in the ectodomain at positions 38–40 (NKT), 554–556 (NAS), and 592–594 (NIS) using NetNGlyc 1.0 (http://www.cbs.dtu.dk/services/NetNGlyc).

Between the G and L genes, a complex intergenic region was present that contained 3 ORFs of 246 nt (7,413–7,658 aa), 231 nt (7,716–7,946 aa), and 459 nt (7,893–8,355 aa), of which 2 were overlapping frames (U1–3). Additional ORFs between G and L genes were detected previously in other rhabdoviruses (*8*,*9*). We detected transmembrane domains in the amino acid sequences of all 3 additional ORFs, suggesting they might act as viroporin (*8*,*9*).

The L gene of RFFRV contained 6,591 nt (2,197 aa). We detected several conserved domains and motifs, including RNA-dependent RNA polymerase, mRNA-capping region, mRNA capping enzyme, and virus-capping methyltransferase. Alignment of the deduced amino acid sequence of the L gene with the L gene of various other viruses belonging to the Mononegavirales order by using MAFFT version 7 (http://mafft.cbrc.jp/alignment/software/) and subsequent phylogenetic reconstruction by using a maximum-likelihood tree (WAG+F+I+G model with 100 bootstrap replicates in MEGA5 [http://www.megasoftware.net]) suggested that this virus belongs to a novel genus of the *Rhabdoviridiae* family. In addition, pairwise identities of the deduced amino acid sequence of the L gene of RFFRV with that of other rhabdoviruses of the *Rhabdoviridae* family were only <35% (Technical Appendix Table 2).

Because the fox was found dead and no tissue samples were collected, whether RFFRV played a role in the animal’s death is unknown. In addition, multiple attempts to isolate this virus on various cell lines of eukaryotes (Vero E6, MDCK, CRFK, N2a, and BHK cells, primary fox kidney cells) failed because of the absence of cytopathic effects and viral replication by quantitative reverse transcription PCR, despite a high number of reads in the original sample. The fox might have acquired the virus through spillover from a small prey, such as a bat, and additional studies are required to elucidate the prevalence, original host, and pathogenic potential of this novel virus.

Technical Appendix Characteristics of red fox fecal rhabdovirus (RFFRV) genes and intergenic sequences; pairwise amino acid identities between the L protein of RFFRV and other rhabdoviruses; RFFRV partial genome; and deduced amino acid sequences RFFRV genes.

## References

[1] International Committee on Taxonomy of Viruses. Virus taxonomy: 2013 release [cited 2014 Feb 9]. http://www.ictvonline.org/virusTaxonomy.asp

[2] Matha IS, Salunke SR. Immunogenicity of purified Vero cell rabies vaccine used in the treatment of fox-bite victims in India. Clin Infect Dis. 2005;40:611–3. 10.1086/427700 15712086

[3] Bodewes R, Ruiz-Gonzalez A, Schapendonk CM, van den Brand JM, Osterhaus AD, Smits SL. Viral metagenomic analysis of feces of wild small carnivores. Virol J. 2014;11:89. 10.1186/1743-422X-11-89 24886057 PMC4030737

[4] Palacios G, Forrester NL, Savji N, Travassos da Rosa AP, Guzman H, Detoy K, Characterization of Farmington virus, a novel virus from birds that is distantly related to members of the family *Rhabdoviridae.* Virol J. 2013;10:219. 10.1186/1743-422X-10-219 23816310 PMC3722107

[5] Albertini AA, Ruigrok RW, Blondel D. Rabies virus transcription and replication. Adv Virus Res. 2011;79:1–22. 10.1016/B978-0-12-387040-7.00001-9 21601039

[6] Coll JM. The glycoprotein G of rhabdoviruses. Arch Virol. 1995;140:827–51. 10.1007/BF01314961 7605197

[7] Käll L, Krogh A. Sonnhammer EL. A combined transmembrane topology and signal peptide prediction method. J Mol Biol. 2004;338:1027–36. 10.1016/j.jmb.2004.03.016 15111065

[8] Gubala AJ, Proll DF, Barnard RT, Cowled CJ, Crameri SG, Hyatt AD, Genomic characterisation of Wongabel virus reveals novel genes within the *Rhabdoviridae.* Virology. 2008;376:13–23. 10.1016/j.virol.2008.03.004 18436275

[9] McWilliam SM, Kongsuwan K, Cowley JA, Byrne KA, Walker PJ. Genome organization and transcription strategy in the complex GNS-L intergenic region of bovine ephemeral fever rhabdovirus. J Gen Virol. 1997;78:1309–17 . 9191923 10.1099/0022-1317-78-6-1309

