# Supplementary material for: Novel Divergent Rhabdovirus in Feces of Red Fox, Spain
Source: Emerg Infect Dis. 2014 Dec;20(12):2172–4. doi: 10.3201/eid2012.140236 (PMC4257811; doi:10.3201/eid2012.140236)
Supplement: Technical Appendix — Characteristics of red fox fecal rhabdovirus (RFFRV) genes and intergenic sequences; pairwise amino acid identities between the L protein of RFFRV and other rhabdoviruses; RFFRV partial genome; and deduced amino acid sequences RFFRV genes. [file 14-0236-Techapp-s1.pdf]

Article DOI: http://dx.doi.org/10.3201/eid2012.140236

Novel Divergent Rhabdovirus in Feces of Red Fox, Spain

Technical Appendix

Technical Appendix Table 1. Characteristics of RFFRV genes and intergenic sequences*

Gene	Length, nt	Length, aa	Conserved intergenic sequence between 2 ORFs†
N	1,629	542	UAG-60nt-ACAAAAAAACUAACCUCAGCUAUG
P	2,490	829	UAA-75nt-UCAAAAAAACUAACACAAGCUGCGAAUAUG
M	813	270	UAA-23nt-ACAAAAAAACUAACCUAAACUAGGUUAUAAUG
G	2,010	669	UAA-70nt-ACAAAAAAACUAACACAAGCUAUG
U1	246	81	UAA-29nt-ACAAAAAAACUAACACUACCUCGUGUUUAUG
U2	231	76	NA
U3	459	152	UAA-116nt-ACAAAAAAACUAACACUAUCUGUGUUAUACAAACAUG
L	6,591	2,196	NA

*NA, not applicable; ORF, open reading frame; RFFRV, red fox fecal rhabdovirus.
†Start and stopcodon of the flanking genes are underlined.

Technical Appendix Table 2. Pairwise amino acid identities between the L protein of RFFRV and other rhabdoviruses

	RFFRV	LNYV	BEFV	RABV	IHNV	RYSV	PRV	SIGMAV	VSIV	TBIV	BASV	FARV	LBV	SHIBV	OZEV
RFFRV		27	31	34	23	27	33	30	32	31	30	29	35	35	35
LNYV NC_007642	27		25	30	23	35	25	25	25	25	25	33	30	30	30
BEFV NC_002526	31	25		45	24	24	60	57	60	57	52	25	46	46	45
RABV SRV9 AAT48626	34	30	45		25	28	46	44	46	44	40	29	86	87	88
IHNV L40883	23	23	24	25		22	24	24	23	23	20	27	25	26	25
RYSV NC_003746	27	35	24	28	22		25	24	25	23	24	30	28	29	28
PRV HM566195	33	25	60	46	24	25		60	74	54	50	26	47	47	47
SIGMAV Q410979	30	25	57	44	24	24	60		59	52	48	23	46	45	45
VSIV AAA48441	32	25	60	46	23	25	74	59		53	51	27	47	46	46
TIBV GQ294472	31	25	57	44	23	23	54	52	53		52	24	43	43	42
BASV JX297815	30	25	52	40	20	24	50	48	51	52		23	41	41	41
FARV HM627182	29	33	25	29	27	30	26	23	27	24	23		28	28	28
LBV JX901139	35	30	46	86	25	28	47	46	47	43	41	28		92	86
SHIBV ADD84511	35	30	46	87	26	29	47	45	46	43	41	28	92		87
OZEV FJ905105	35	30	45	88	25	28	47	45	46	42	41	28	86	87	

Red Fox Fecal Rhabdovirus, Partial Genome (GenBank Accession No. KF823814)*

GGATATCAAGTCCACCAATACCTTATATGTGCATGATCATGCACATCACATTCTACG

CATACGACTCCCAGGGAGTACACTAAAAAAGACGTAACACAGACTTTGAATTACGT

CAAGTCTAAGTTTTAAATTCGGTTAATTTCAATGGATCACGATAACGAAAAGCCAAT

CTCGTACACTTCAATAGCGGAAGTTCCTGATAATGTTGCCATTGGGAGCACCATTTA

CATTCAAGGTGAGCCCATCATCTATTTTGGAAAATCCGCTGCAACAGGAATTACGCG

GAAAGGGGGGGCACAGAAAGACTGGACCAAAGACATGATCCGTGGAGTGAGAGTG

TTCCTGCCCCAGACTGATGCTAATCTGCTCAATCTCATAGCCGGGGAAACCGAAGCC

CCTGAGCTGGAGAAGTACACCATCCAAGATCCGGAAAAGAAGGGCATCTTGAAGAA

ATTTGAAAGCAAGTGGGAGTTTGCGAATTGGGCAAACCTGCTGGTCGACTTGCAAA

GCAACACAGGGAACATCCCCAAAGGGAGATTCCCATACTACTCTGCTCTATTCTCGA
TAACTGCCATTAAGGGAGCCCCTGTCCTGGCCCCTGCCATGAAAGACCTCGGGGACC
CTGTTTATGTGAAAGCTCCCGATGACCTCCACCCACCTACAGGAGACATAGAATGGC
ATGGTGATAAAATTAGTGTCGACGAGGCAGCCTACATAGGATATGGAGCATGGCTG
ATCATGCCTAGATTCACTATCAAAGCTGAATCCAAGAAAGATGAAATTGCAGCCAG
CAGCAAGGCATTTGACACTCTTAGGCGGTTGCTACCTGAGATCACCAAGCCACAAGT
GTTGGTATCTGTGGTGACGCAGCTCAGATTGGCATATCACGGAACGCTGGTTCCCGG
GTCTGCGTACCTTGCTGCAGAAGTAGCAATGAGAAGGGCAATGAATATAGAGTATG
ACCTAAAGGCTGACAGAACGGAGTGCAAAGCCGGGGAACACTTTCCAGGTTGTCAA
TTAAGAGTCCTGCAGGATATCCCCCAATACGACTCAGGCTTTTGGGGCTTTGGGCAA
GTTGGGCTAGAAATGGCCGGATACTCTGCTCTTAATATGCTGCATGCCGGCCTGGAC
ATCTACGGGAAAACCATTGCTGACCTAAGGATGCTAATCAACTGGAGGTGTTACGA
CAACTACATCGCAGATGAGATTAAAGAAGGCCCTTTGTTAGCAGATGACCCCTGGA
GAGCAGCGTCTTACTTACTAGCCCCTAATATAAGAACGCCACTAAGCATGGGGAAG
CACTCCATTGTAGCGTATTTGGGGCTATCTATCCAGTCAGCTGCAGCTAACATCAGC
ACAGGGGCTCCATCCCCACCAGAGGGAGTGAAAATGAACGAGCTGATCAGAAAGA
CAGTGTACGATCATGCTGTTGCAATTGTTAGCGAGTGGGACAATGACAGGCTTCAAC
CAAGCACCGTCACCACTGTAATGATTGGAGGACAAGTTATCCCCTTCAAGGGAGTTG
ACCCTAAGCGAGTGAATGACTTGTCAAGAATGTTCACTCAGAGACAGACCCCTTTGT
ATGAAGTACCACCCCACAATCAGAGGAGGGAGCGATCACCCTCTGTTTCCAGCGTC
CATACTAGTAGTAGAaGAGATGaTGAAGGGAGCTGGGAAGGAGGCAACGAAGAAGA
GTTGCTGAGAAAACTCCATGAAAGGCGAGGCCAGTACGAGGAAGATACTAATTTAG
GGGGATTTTATTCAGCGACATAGACATCATCTTGAAATATTATAAGCTTGCTTTTTGA
TTCTTTAGCTTAGTATTAGTGCCATACAAAAAAACTAACCTCAGCTATGGCTAATCG
TGTAAGGAAAATTCATTCTGTTGTGTCCGTGAATGATCCGGCCGAAGCTTGGCAAGT
TGCTCTTCAATATTGGGCTAAGCGGATCTTGTCAACAGATAAGGAGCTGCTCTGCCG
AATTACGAAGTCAATGGTTGCCCACATGTCTGTGGTGTACGGGAACGTTAAACTCGA
CTGCGACGCCGTTTGGAGAGTCTGGGCCCTCCTTGCATTAGCATGGATGAAGAGCCC
TGTAGACGGAATTGCCATGTTGGCCTTACTAGAATTTGGCGCCAAACACGTACAGAG
GCTGCGAGCCGACCTGAGCAAGATCGGAGACCCTAGAATCGATGATTTCGAGCGGT
TAACAAATACCAGCATTGCCCGTGTTCCAAAGAAATTCTTCAAAGAAAACAGCATC

ACTGACGACACCAAGAAGAAGCTAGAAAAGGCTGCAGCAATCTTGTCGGATCTAGG
GAAGAAGTGCAGCTATACTGGATTTTGCTGCTATGCGGATGATAGCAACGTAGGAG
ATATGATGTCGCGGGTGTTAACGGCTTCTGGGGCATTGGGATCTGAGGATCCTATTG
GTGTGTGGAAGCCGTTGGAGTTAGATGAGAAAGCACCGTATCTTTTTGATGAAGTGA
AGCGCATTGGAGGAATCTCTGCAGAAAAACCAAGCCCACCTGAAAAGAAGATGTGC
AGGTTATTCGGGAAGAAAGGGAAATTTGCAGCGCCCGAGACAAGCTATAAGAGTGC
GCACCCGTTCTCTACTCAAGTATCTTCCAAAGAACCACCAAAGTTGAAGGTCCGAGG
CGTGGAACTCCCATCTCTTGATGAAGTGATCGGTCTTCTGCCCAAGGGAGCAGAGGC
TGCAGAACTTCTGGACGACACCAAGGAGTTGGAAGGCGCATCAGCTGAACCAGAGC
TAGCCGATCATGCCGAGCCGCAGCTGACTCAGTCACGCTCTGATTATCAGGGAGATC
AGAATTTCACTTCTAGCGTGCTCAAGGCTAGTCCCCCACATGGACACAGTATAGACT
GCGACTCTAGCCAAATCGGGGCATCAAACCTTAGCGCTAGGAGCGCAAGCCTCTCC
CCCGAATGGAAGGAACCGGATGGAGCCACCAAGGGCGATGATCCTGAAGAGGTAG
AGTGCGCCAACGAGATAGTTGGAGGAAATAGAAGGGTTAGCGAAGAAGGCTGCTGC
TTGAATGAAGACACCAGAGAAGAAGGCAGCCTTCAATCTGATCAACACCCTGTGAA
AAATTACAGTAGCTGGGCAGAGTTCCGAGCGTCCTTGGAATCAGAAGTCTCTCCCAA
GCACGCGGAGGCTGCTGGATCGGCAATAGGCCACTCCCATAGCCCACAAGCAGAGG
CAGTTATGGATTCTGTAGGAGTTGCTTCTTCTACCATCAAACCTCTAAAGTCCGGAG
TAACGAGGCCTATGCAGACAGATGAGCCGCATGCCATCCTGGAAGCAGGATCGCAG
GGTATTACCAGCCTGTACCCTCCCCTCCCAGTTGTACCCGGAGTATTACTGCCGAGT
TCTCTCAAAAGCTCCCTCCCCCCGAAGGCGAAAGGGAAATTCACCGAAGAGTTCGG
ACTGATGTTACGCGGGATACGAAGAGGGCTAGAAGAAAGAGGCCTCAATTGGGAGG
CCAAATGGTTTGACACGCATCTGGAAGGCATTGCAAAGTTTGGCGACCCCGATACTA
TTGGGTTTCAGCTAGGTAGCTACTTTGCTCAACTCATTACAAATCCTTACCTTAAGAG
GGAGGAAACAAAACCGTCACCAAAAGAGGATCACGCGCCTCCACCTCCATCGGCAC
CTCGAAAAGAAAGCGTGAATAAATTCAGTCAAGCTAGGCCCCATAGCCAAGTGCCC
ACCAAAGAAGATCCTGCACCGCACCCTCCTAGAGCAGAAGAACCCGACCCCAGAAG
CTGGATGACTGGGAAACCATCAAGGGTATATCTGCCAGGATCGGATGACGTTATGG
AATTTCGCACCAATGAGGCGGAGCTTGACAACTTCATTACATCATGTGTGCTGGAGG
ACAAGTTCACTGAGCCATATATCCTTAAGCCTAAGGAGTTGTCCAAAGAACAGCTAA
AGAATCTCTTAGAGGTTGTGTCACAACATGGACAGAAGGCTTCCCAGCTGTTATGTG

AACATTTGACATTAAGAAACTACAAGAGCATCTCAGCTTTGACAGCTAATTGGGCAA
TGAAGATAAAAGCAGAGATGCCAAGGTCCATGGGAATATACTTCACAAGGCTAGGA
GAAGCATATGGAAATGCCTTCTGGGAGGCGCTGCAAGTTGCTACTGCTGGCAGAGA
AGGGATTGACAAGTGGATCAGGGCTGCACTAGTCGTGAAGGCTAACACTGCGAGAG
GGAAAGTTCCTCCCCCTGGCTGGTTTGTGACAACAAGAGCAAAAGACAGCGACAAC
CCCAGCGTCCGTGCAAGAGCAAGTGCGCTGTTTAAATTCTTAAATGAATAATATTAG
TCTAACCCCCTGTAGTTGTAGTCTTGCCTCTATATTAGCTTACAAGTAGTTCTTCATC
TCATCTTCATTTCAAAAAAACTAACACAAGCTGCGAATATGAGCAATTTTAGGTATA
CTCTGATGAAATTTGCATCAAAGATGTCTTTGACATCTAAATCTAAGTATAAGGTAC
TTGGAATTGGTGACGAATTGGGTCAATCGAATGTCAACATTATCCATGAGGGCGAG
GAAGACCATACGTCGATTTATTCAGAGAGCCCTTCATCTTCTAAAAAGAAAACAAGT
CGCACAATGAGGCCTTGGGTTTATCCTACATATCCTGAGCAAAAGAACATAGTTAGA
GGAACACTGCGCGGAGCCAGGCCTAAGAAAGTTGTTCTGTCTATTAACTTAACTGGA
GTCTCTGAGCCTCTTGATTTTCCGGAAGTGGCCACAGTCATATCAGACATTCTTGAC
GGAATGGAAATGTCTGCAATGAGAAAGCTGGTCTTGCAAATGATGATCTTGTCAACT
AGACCAATGGGGAGGTTTTCTGATGGGTGTTACCGATACATCTTCAGTAATTGTTTT
AGCACCTCATGCTTTCCGTCTAGTTGCCTAGGAGAGTTCATGCATGATTCAGGGGAT
TTCTGTTCGACCACGTTTGATGACAAGACATACTTTGGATCATACAGGCTGACATTC
GAAGACGCGATAGGCTCCAATCATCAATACCCGCTCCCCTTGTGGTTTCACTTGTCTT
ACACCAACGCGCTCAAGCCAGACTTCACCCCCGGACTAGCAGTCTGCAGAGCTAAA
GGGAAGTTCTTGATCTACCTTGATGACAAATACTTCAGCTTCAAAACGTATAGCGAC
AACATCGTGCTATTGCTGATGGGCCGTGCTCCTAAGGCAATTAAGCAGAAGTAATTA
CCGCTTGCAGTTTGGATTTTACAAAAAAACTAACCTAAACTAGGTTATAATGTATCA
TCTAATTGTTCTCCTTGTTATGCTAGGACAAAGAGCAGTAGCAGAGCCAGCTGACAT
TGCACGAGCCGCCAAGATCATACAGTATGCAGAGGAAAGGCTGAGAAATAAGACG
GGTCTGAGGATTGAGCTGTCATCGAGAGATCCGGCTCAAAGCGCAGCATCCCCACC
TGATGTGCCTGCGACTAAGCAGCCTGCTACCAAGCCCGCTGCTACTACAGCTGCCGG
AACTTCAGCCCCGATTAAGGTGGGGTTGCCTGAGTCTGAATACCTGCGTCAGGTTGT
GGAGCCTTGGTTCACCGTCTGTGAGAGCAAGGTCCCAAGGACACTGACCGATGTAG
TGCATCGAATCCTCAGGAAAGGGGATGGCAAAGCTCATAGAATGCTTCGAGGAACA
CCCAATGGAGATGATCACTCCGTTTATACAAAATTCGGATCCGGGATCTCCCCTGCA

CCAAGCCGACTGGTAGTCCTTAGTGAATCATTGCAAGAAGTGCAAAGGCCGCAACT
ATATCGAGTCTCGAGGCTAAGGTCAAGCTCTACGCCCCACCGGGTAGTACTATGCGA
AGTTATGTCTGCATTCTCACCTCTGAATGTCGAAGAGATGGAGTGCACGGGAGTACT
GGGTAAGCTGTCTGACACGTCCTCGGTTACGCTCAATGTGGTGGACCCGGGGTATGT
ACTGACAATGATCCGCGTCTGCTCTTGCTCAGCAATGAGGGTCAAACGATGGACCTG
GACTAGCTTCTTCAACGTGAAAGAGAGGCTAGAAGACCGCTGGAGGATACCCCCTG
ACCCTAAAGAATGTGAGTCTCATTGCGAACCAATTTTCGCAGGAGAAGTGACTTCGG
CTGTAAGAGGGAGTGTTCCTGATTATTGCTATGCTTGGATGTCTACGTGTGAAGTTC
AGGGAGATGTGTACCAAGTCTCTTTAGGAACAGCTAAATTCCATAGGTTCTTGAACC
AAATAAGAGCCCCCTTTATTGTGGACGACCCTTGCACCCCATCGGCGCCATGTAAAG
GATCAGGGGATAGTCTCGTCTTAGCAAAAATAGAAGAGGATAACCCTCGATTCACC
ACCATGAATGGGGAATTGACGCCAAGGTACAATTGGGATACGCACAAAGTTTATCA
GGTGCACCTGCCAGGAGTCACAACTAGCATCTTGGACGCGTCATGCGGTTTTCTACA
TGGGGGTTATGTGTATTATCAGCTAATGAGCGGCCGCATCGTTAGCGTGAGCGTAGG
CACGCTACAATTTGGTGATAAGGTCAAGCCTCCGATCTGCACAGAATGGAAGGGGC
CGTATATGCCGTTTGTCTTACCTGATTCTCAGGTTGCCTCGACTTCTGAACAGTTGCG
GCAGGACCTGCTCCACTGCCAGACTAGAAAAGAAGTCGTCCTTAATGCACTGGCAA
CTAAGAGGCTCCCATCTATAACGCTGTTTGAAGGCTTGGGTTACAAAGGATCGGAGT
CATACGGTCTGGTGTCAAGGAAGGGGCTCCTGTATGCAGCACCTTGCCCATCAGTAG
AGTACACAGATCTTGAGCATGTAGAAGGAAACATATGGATGGTCGTAAATAATGGG
AGAGAAGTTGGGTGCCTGGATGGAGGGTTAAACTTTGCTGTCAAGAGCGGTTGCGT
GGTTAATCCGAACGCATCCGTCTCTATCCTTCTAGGCGAGTGGAAGGTAATATCTGA
TAGGGACGGCAAACTCTTAGCAGAACCAATCCCAAAGGCAGGTTGGGGGAGCATTC
CAGCTCTCGAAAACATATCGGCTGCATTCGGGGATTACTTAGCCAGTTTAGAACAGC
CGCCTCTGTGGGATGATGGGAACGGGCCCATTATCATTCCTACTAGCAACTCAACAG
GAGACCCGGTCATTCATTCCGGGGCATCATCACTGTGGAGCTCTATGTCCCTAGCCA
GTAAAATCACTGCTATTCTGATGCCTCTTTTATCCTTAGCAGTAGTGGTAGGGATAAT
AATGTGCTGCCGCAGATAACTTGCATATCATGTACAAGAAAGATATTGTTACCTTCT
CTCTGATAGCTTTTAGTTAATCAAATTAAAAAACAAAAAAACTAACACAAGCTATGA
GTTGTTTAACTCAAGATTTGAGGAAGGAGATACGAGAGCGGGTGTCTAATAGCATA
AGCCAGAATCCAAGTGTAACAACACTTATAATTATCGAAGTTATACTATGGGTCGGC

ACTTTAGCCTATATTTCGATAGCGTTGGGGTGCCATCGGTATCTTCAGGCTCGCATA
AAGAATTCTGTAGAACGGAGACAACGATTTAGGGGAGATGGGGTCTTGCTGCCCGA
TACTGGACCTGCGTAATAACTAAATAGGTCGATCCTTTAGGTGGTACAAAAAAACTA
ACACTACCTCGTGTTTATGGAAGAAAAGTGCTCAGATTCCGATTACTTTAAAGAGTT
GACTGACGCAGCTATAGAGGGAGTGTGGGCATCTCCTTTATACCCAATTACATTGGC
TACGGTCATAATATTCCTTATACTGCTAATATTTGTAGTAGCGTGGAGAGCAGCTGT
GATTGCAAAGATACGACATCGTATAG*ATGAGTCCACAGCACGGCGCCTCCGCCCTGAT*
*TTCGGGGGTCGACCCTG*ATCACTTCTCGCTTATCATTGAGTCATACCCGATTCTTGTG
GGCAATTATGCAGCCACACTCATCTTAACTGTCACCCTAGTCTTGTCTTTTAGTCTTG
CTACAATTATTTTCTTTTTTTCATCTGTAAACAACTTAACGGATATCCGAGGCGTTCT
CATCTATCACTTAGGTAATGAGGTCTCCGAATTTGCGTCCCATGCTTTAGCAGCAGTT
TGTAGCGCACAATCGACATCTGCCAGACTAACAAGGGAGCTTGAACAGTTTCGCCTA
AGTGGCCAAGTGACCGAAGTAACACCGAGCTCGGGGGCAACGGGCGAGCTGAGCA
TACCACAGCGCTACAGAATGCTGTTAGTAGAAAAGGACATGTTGGAAAATGAAATG
TTCATTGCAGAGCATTCGCTAGTCTAATCTCTAACAACCTACTTTGATTTCTTCATCC
TCTCTTGTCTTATGTTTGTGAACTGCTTTGACTCAGTGATTTCTCACATAACAGCCTA
AATATAACCGCAAATTATCTCTCAAAAACAAAAAAACTAACACTATCTGTGTTATAC
AAACATGGAAAAATCGTTCCTTTTTGATCGCATTCCGGACTCTGTGCTGCACAGCCC
CCTTATCCTTAGAGAGGAGTTGTGCTTTTGCCGTCCAGACAAGGAAACCAGTGACTA
TCCAGCCATTCCGCAGCCAGACGAATTCACAATCTCTCGCCGCCCTGAGTTGATAGA
GCTCAGGTCACTGTCACTCACTGGGGCAATCTCAGGAGTAGGGTGTATCAAATGGCT
CGCTTATATGAGATTGAATTGGATCGAAGCCAAGGATATTATGAGGCTCATGGCTGA
AGAATACCAATCATCCACAATCCACCCAGAGATAGCATCCGACTTCAATAACGTGT
GTGAAGGTCTCTTCTCAAGGCTGCATGACCAGTGGGGAGAAAATCAGCATCCCAGT
GCGCTTCAGGACATTTGGAACGTTTTTGTGGCGCAGTACCAATCAGACGCCATTATT
GACAACTGGACAAAATACCGAATACTCTCAGCAGCTCTCTTATGGTCTAATCATAAA
GAGAGAGGCAACCAATCAGAATTTCAAAACAGCATCATTGCTAAAGCAGGATGGGG
AGTCCGCGCTATAAATGGAGTGGAAGTCATAACTCACAAGTTGTTAACTAAAAGAG
AACGCTTGGGAAAGCTAACTGTAGGCGCATACTGGTGTCTGCTGGAGCTTGCCAACC
AGCCTCCCAGATTGTTCAGAAAATCACTATTGCTGTGTGAAAAGGACCTGTATTTGC
AGCGCTGGATTTCACTCTTCTTGTGTAACTGTCAAAACGCCTTCGGAGGAATCCCTTT

GCCTCAGCCAAAGCACTTGATCAAAATGTACCGAATGGGTGACATCATCCTCGCCAC
AGAAGGAAACGAAGGGTACAATGCAATCAAGACAATAGAAAGCATCATGGTCGGTT
CTGCACATAAGCAACTAGAAGACGAGTCAATTTCCCCTACAGGTGACAAATTCTACG
AGTTTATCACGAAAGAAGGGCTTTCAGCTTGCCATCTAGAATCATCGTCATCTGCTA
TGAGAAAAATCATCCATATAGGGGAGATAGTAACCCCTGAAGTTGCCATAGAGATG
GCAGGATGCTACAGACACTGGGGCCACCCAATCATCAATCCTGTTGGAGGTCTCACT
GCAGTCCGAGAGAACGCAACTGCCCAGCTTCCTACGAATGAAAGGCTCATGATATC
ACTAGCTGCTGATCTCAACTATTTGTTGTTGAGGAGTTATTTTGAAGAGCATGGCAG
GTGGCCCCCGGGAGTGAGATATGAAGCGGCGCCAGAGATAGCCAAAGACCTGTTCA
ACAAGTGGGTATCAACTAATCAATTCCCTGGCCCTACCTCTGCTAGTCAAGTCAGGA
ACAGCTGGTTTTTCGTAACATATGACAGCTTATTCGACAAAAATCAAGAAATTCCAA
TCCTCAGCCTAATTTCAGACAAGTCTCACTCAGTTGGACGATCCGCCCTGTCAACTA
TGTGCCTCAAAAAGAATCTGCTGCTGAGTCCTTCACGGAGGGTGCTTCAATCAACTC
TCAGCTATGCAGAGATCGATGTGAACAAATTTTTGGACTCTATAGACAGCACAGAA
AATGGTTTAAGCAACGACGACTTAGTCATCCTATTGCGAGAAAAaGAGCGAGAGCTC
AAGGTAAAGGGGCGGTTCTTTTCCCTAATGACGTACAAATTGAGGACTTACTTTACA
GCAACCGAATACCTAATTGCAAAGCACATTCTGCCTGTACTCCCTGAAATTACAATG
ATGCAGGGGCAAATTGACCTGTGGAAGACTTTCAAAGGTGCGGTACGCACTGTGAG
TCAAGAGAAGTCTACACATCACATGATTCATGTAGATTTCGAAAAATGGAACAACTT
CCAGAGAGAAGAGTCTACAGCTCCGGTTTTCCAGATTATGGACAGAGCCTTCGGGTG
GTCAAATGTCATATCAAGGACTCATAACTTCTTCTCTAGGTGCTTCGTCGGCTATGCT
GGGAGAATAGATATGTTCCCTATCGGTCTGACAGACAACTGGCCGTGGTGTTGGACC
GGACACAAAGGAGGCTTTGAAGGGCTAAGACAGAAGGGATGGAGTGTCGTAGGGG
CTTTGCTAATACGGCACGTGATGAGACTCACTGGTCTGCATGGGAAAGTGCTAATTC
AAGGAGATAACCAGGTAATCATACTTGAGTACCCTCTTGCCAGCTCATCGAATACTT
CATCGCTTGCTCTCGAGAGGCATAGGCATTCGAAAATGACAACTCACTTCTTGTCTG
TGTTCTCTGAATTGTCTAAGAGCATCGGATTAAGAATTAAGCCAGAAGAGACATGG
ATATCCTCGAGGATGGTTTACTACGGGAAGTTCCCTGTGATCGACGGATCGGCAAGA
GGAATGGTGCTTAAGAAACTCTGCAGGATCTTTGCAATATCAAATGACCTCACTCCT
AGCATCTCAAATAGCATCTCTTCTCTGCACACGTCATGCATTGCCGGCTGTATCCAAT
CATCATACGCTGTCCCAATTTACATGTTTGCTATCTTTTATGGCTTCTTCCTGCTCCAC

GACTTCTTCGAATACAACCCCTTGTCCATGGAGCCTATGATCTTCACATTCAGACGG
GCTTTATTCGGAACAACTGCTGTTCGAGACATTACCAAGGTACGAGCGCCCACAACC
AAGGACTCTATCATCATTTCTGTAGCAAGAGACTGGGCACCAGCAAGCAATAAGCA
GCAATCGGCTCTCGGACTAGACCTCCTGACGCGAGATTCATCTCTTGGAGGTCTTGG
AGGAGCATCCATTTTAAAGTATATGATCAGGCAGTTTCCTGATCCTGTCACAGAGGG
CCTATCATTCGCAAAAGTGGGGTTAAGCAAGGCAGAAACACCTGAGCTAAAGACCA
TTTTCTCTTCTTATGGAGGCCTTCCCATCAAGTCAGGCTCAGTAGAACTCCTTCTAGA
AGCACCCATGTCGATCAATCTGCCATCCTCTACTCGTCCTGTAAATGTACTGCGGCG
GTTTGTTGAAGAGAAGATCACACAGAACATGGACACGCTGGTCAACAATCAGCTTG
TGTATCAGGCGCTGTCGATGAAGCAAGATATGCGACCGCCATTCTTGAAGATGTGTG
CATCCCTACAGCCATTCTTCCCCCGATTTGCTTCTTCTCTGTACAGTGCCACCCCCAT
AGGAGTGGCAGAAACAATTGTTGGAAAATTCCTCGGGACTAAAACTGTTGCCAGAA
TTGCTCTCAGAGAGGGTGCAGGGCAGCTGGGAAAGAAACTAGTAGAAAGCGAAGTT
GAGTTGATAACAACGTATCTAACAGTTCTCAAACTGTCTGCTCTGGAGCATTGGGGT
TGTTCTAGCAAATTGGCTGACAGCTATAGGTCGAAAAGTTGGGGAGGCCAAATTTG
GGGAGTCACAGTGCCTCACCCTGCAGAGCAGTTTTACTTAGAGTGGCCAATGACAA
GCGGGTGCTTCAAGGAAAGCCTTGCTTGGATGAATGATTTTGCCGAAGaAACTGTGA
AAGTTAGCTTGAATGAAaGAATGATCTATGGAGACACCTCTCCGCGATCAGCCGGAC
CTATGAATCCTTACTTAGGTGGTCGAACTAGTGAGCGGTCCTCTGTCCAATCATGGC
TAGAGCTCGAAACATCATCTTCTATTGCAAAGAGACTTGTAGAGCTAATAAATGCAT
GTGGTTGGGCATATCAAAAGGGTTCGCACTTGCATCACGCAATAAAAGAAATGCTC
ACACAATTGACTGGATTAGTGATTCCGAGCGATGCAGTTTGTAGAATCACAGAGTCC
GGGAGTTTGGGACATAGGTTCTCAGATCCTAGAGTGTCTTCTGGAGCAATGTCAGCC
ACCAACTATAACACTGCCACTCATATTGCGTTCAGTACAAACGACCTAGTGAGGTTG
AAGCGCGGGGAGGACAACTATATGGTTTTGTTCCAAGGGCTATTCGTGTATTTCACA
AGCATTATTGGGGAGCTAGTAAGGAGAGGAGATAATGGGTCTCCGACTGCCCACCT
TCACCCTAAATGCGAAGAATGCATGATACCATGTCCAGATGTTTCAATGGAGCTTTC
AAACCCGGTAGTCCCGCTTTATTCAATGTTTTTATCACCTCGCCTCCCGAATGATTTC
TTAACGTTGCCGGAGAATCTCTATGTTGACATTGATCCGTTCAAAGTAAAGCCTGAT
CAAATCAAGACTATTGTCAACATCGTTCCTGCATACAGTACAGTCTCTGTTGACACC
CTCTCGGGTGAGGAGATATCAGATGCTATATCCAATGTTGCAGCAATCATAGCAGTA

ACAAAAACAAATCCTCAAGGAAGATGGACTCTGGGGATACGAACTGCCATCCCGGT
GTATGCAGGTTACAAGTGCTCGTGGAAGGTACTTAAGAGCAAGATTGTATTCTGGAC
TTTTGCTATGCTTGCATTCCGGCACATCAATGAGTCAGAGGAGCATGACTGCTGTGA
GGCTTGTCAGCACATACTTAGAAAGCAAGCAGCAGATCACTGGAACCTGCATAACC
CAACAATGCTGGGAGGGATGCTGAGCAACACGGACATCATTGTCGAGTTGGCTAAA
GAGCAGATTGCGCTCAGCGCAGGATTTCCGATATCGCCTATGGAACTTTCAAGCGCA
GCAGGACAGTCATTATTCGAGCAGTGTGTCTCCCATGGAAAAGAATTCTCACTACCA
GAATTCCCGAAGGGAGTGATAGCTCTCCCGAGAGACATGACAGGAGGTGGAACTCC
GGCATATTTAATCGCCTGCATGATGTGCTATGCCGGCTTCTTCAAAGGAGCCAACAC
GGGACCACTAAAAGCACTTGCAGGGCTGACCACTGAGATGAAAACATCTGTTATAG
CAAGCCAAGCATTCGAAGAAAGCGAAGGAGTCTGGAGCAGGCTTGTAGATATGCTT
CACGAAAACACATCAACCTTGTGCTGCGTGCTTGACATTGACTTCAAAACACTAACA
AAAAGGGGTCAAGACGTAGATCATATTCTCCCGGGAAGCATACCTGCTATTTCACTT
GATACCGTCAGAGGGGCATACTGTATATGGAACACAGCATCAACAACAAAAGGGCC
TGAATCTTCTTACTTGGAAGAGGAGGGAGAGCCTGAGGTGACACAAACCCCTATCA
TGCATCTATTCCGCCCCATCTTAATGTCTGCTAACGGTTCAATCAAGATTCTGTCTGT
CTTGAAAACTATCCAGCTTCAACCAAAAAATGTTGCAGTGGTCGGAGATGGTAATG
GGGGATTTTTGAGGGCACTATTGGGTCTCTACCCTACAGCTGAGTTCTTTTTCAATTC
CCTATCTGAAATGTCCAACTACGGCGATCAAGGGGAGTTCACAAATTACCCCGCTTG
TGTGCTAGACTCTCCAAGTGATTGGGGAAGGGTGATCAATCTCGGAGAAGTCTTAAC
ACTGCCTTCAGACTTGTCCTCAGAGAGGTGGCCTTCCGAGGTAACGAAATACTGGAT
GAAGAAATGCTTTGACCCAGACCTGCTGGTGTGCGATGCAGAGAGCTATGATTCTAT
CAAGAGGTCAGAAATCTTGAGCAACCTCCTCAAGGTATGCGGCCAGCGGTCTCACG
TCATAATCAAAGCCCACTTGTCAAGGATGCAGGACGTCCAGAGCTTGGTCAATGCGC
ACTTGCATTCACACAATGTGATGTACTTGAGAGCCCCTTACAGCAACTGGGGGAAGT
TTGAGGTGTACTTGTGGTACACTCGAATTGTAGAAAGAAACGATATGAGGTATGCAC
CAAAAGATACATATCTCATGTGGACTAGAGCTGAATTCAACGCTCACCCTCAGCTCC
AATTAACAGCGTTCCACAGCTGGGTTTTCCCTCATCTTGAAAAAGACGATAGACTCC
GAGCTGTCCGGCGGATGCTAAGCCTACGACGGCACATTAGCAGGTACACTAAGATA
AGACCGCAAGAGCTGTCACGAGTAGAGAGGAAAGCAAAAGAGCACGGAGACATGG
GGTTCTGTGAATTGCTGTTGCACTACTTAGGATCCAAGTTCATATCATCTATGAACA

ATTCTGTTATCAGGGCAACTTCACATGCTGGGAGATTGAGAGCCAGCTTAAAGCGAT
CGGAGCTGATCGGGCTAATAGGGACTGCTATCGGTGCCTCATGCTTGGTGTCAGCCA
TTTCTCATCCGCATGATTACTGCTTGTTCTTGGAACTTTACAAGAATGGGGTGGATGT
TGTTTTGGGCAATGATAAAGGGAATATCAAGCCAATTGTGGTCAGAGCCTGGTCAG
GTGAGTCCACTCCTGTAACTATAACCCCAAAGGAACTCAAAAAGCCTGTTCATTCTA
TGGTGAGATACTTGGGGGGCTTATTGTGGAGCTCTGGTATTGATAGAATCACTCAAA
GGTTAGAGTGGCTGGTAAGAGAAAAATATGAACTCGGCTTCTCGGAAGGGTACAAT
CACTTGGCAGTAGCCACTTGGGATAACGATACCTTAGACGTAGATCCATTGGAGTGT
ATAAGATGGGAGATGGAACAAGCTGCCGTCGAAGAAATCGATTTCGGGGGGATAAT
TGATGAATAGCACTTGATTTCTAAAATAGTATCAGAGAAAAACAAAAAACTAAAAA
AACAAAAAAAACATATATACATATAAAATTAGGAGCAATAACTGAAGATCAAAGAG
CATTAAAAGAGATCTAAGAGAAAAAGAGAGAAGGACCTGGCAAAAAGCTAAAAGA
TAGAAATAAGGAAACAAAAGAATTTTCCTGAGAGGGCAGCACATAAGGATGATAGG
GATCACATGAAGGCTTCGGGAACCTTGAAGCTTGTAAAAAAAGCTCAAAGACAAAA
ATTGCAGAGAAGAGGGGAACCCAAGTTGCTCTGATTGCTGACTGGCGACATCTGAC
CATTTGCTCTTTTACTGAAAGACCAATCCTGATATAGATGACTTCTAGTTGTCTGGCT
ATGAGTGTCAGTATCGTACAAGGCATGGGTATGGGGGTATGGAGGGCAGCACCATT
GTGGGT

*Coding sequences of identified open reading frames are underlined; deduced amino acid sequences below, overlapping sequence of open reading frames U2 and U3 gene are in italics.

Deduced Amino Acid Sequences RFFRV Genes

N Gene RFFRV

MDHDNEKPISYTSIAEVPDNVAIGSTIYIQGEPIIYFGKSAATGITRKGGAQKDWTKDMIR
GVRVFLPQTDANLLNLIAGETEAPELEKYTIQDPEKKGILKKFESKWEFANWANLLVDL
QSNTGNIPKGRFPYYSALFSITAIKGAPVLAPAMKDLGDPVYVKAPDDLHPPTGDIEWH
GDKISVDEAAYIGYGAWLIMPRFTIKAESKKDEIAASSKAFDTLRRLLPEITKPQVLVSVV
TQLRLAYHGTLVPGSAYLAAEVAMRRAMNIEYDLKADRTECKAGEHFPGCQLRVLQDI
PQYDSGFWGFGQVGLEMAGYSALNMLHAGLDIYGKTIADLRMLINWRCYDNYIADEIK
EGPLLADDPWRAASYLLAPNIRTPLSMGKHSIVAYLGLSIQSAAANISTGAPSPPEGVKM
NELIRKTVYDHAVAIVSEWDNDRLQPSTVTTVMIGGQVIPFKGVDPKRVNDLSRMFTQR

QTPLYEVPPHNQRRERSPSVSSVHTSSRRDDEGSWEGGNEEELLRKLHERRGQYEEDTN
LGGFYSAT.

P Gene RFFRV

MANRVRKIHSVVSVNDPAEAWQVALQYWAKRILSTDKELLCRITKSMVAHMSVVYGN
VKLDCDAVWRVWALLALAWMKSPVDGIAMLALLEFGAKHVQRLRADLSKIGDPRIDD
FERLTNTSIARVPKKFFKENSITDDTKKKLEKAAAILSDLGKKCSYTGFCCYADDSNVGD
MMSRVLTASGALGSEDPIGVWKPLELDEKAPYLFDEVKRIGGISAEKPSPPEKKMCRLF
GKKGKFAAPETSYKSAHPFSTQVSSKEPPKLKVRGVELPSLDEVIGLLPKGAEAAELLDD
TKELEGASAEPELADHAEPQLTQSRSDYQGDQNFTSSVLKASPPHGHSIDCDSSQIGASN
LSARSASLSPEWKEPDGATKGDDPEEVECANEIVGGNRRVSEEGCCLNEDTREEGSLQS
DQHPVKNYSSWAEFRASLESEVSPKHAEAAGSAIGHSHSPQAEAVMDSVGVASSTIKPL
KSGVTRPMQTDEPHAILEAGSQGITSLYPPLPVVPGVLLPSSLKSSLPPKAKGKFTEEFGL
MLRGIRRGLEERGLNWEAKWFDTHLEGIAKFGDPDTIGFQLGSYFAQLITNPYLKREET
KPSPKEDHAPPPPSAPRKESVNKFSQARPHSQVPTKEDPAPHPPRAEEPDPRSWMTGKPS
RVYLPGSDDVMEFRTNEAELDNFITSCVLEDKFTEPYILKPKELSKEQLKNLLEVVSQHG
QKASQLLCEHLTLRNYKSISALTANWAMKIKAEMPRSMGIYFTRLGEAYGNAFWEALQ
VATAGREGIDKWIRAALVVKANTARGKVPPPGWFVTTRAKDSDNPSVRARASALFKFL
NE.

M Gene RFFRV

MSNFRYTLMKFASKMSLTSKSKYKVLGIGDELGQSNVNIIHEGEEDHTSIYSESPSSSKK
KTSRTMRPWVYPTYPEQKNIVRGTLRGARPKKVVLSINLTGVSEPLDFPEVATVISDILD
GMEMSAMRKLVLQMMILSTRPMGRFSDGCYRYIFSNCFSTSCFPSSCLGEFMHDSGDFC
STTFDDKTYFGSYRLTFEDAIGSNHQYPLPLWFHLSYTNALKPDFTPGLAVCRAKGKFLI
YLDDKYFSFKTYSDNIVLLLMGRAPKAIKQK.

G Gene RFFRV

MYHLIVLLVMLGQRAVAEPADIARAAKIIQYAEERLRNKTGLRIELSSRDPAQSAASPPD
VPATKQPATKPAATTAAGTSAPIKVGLPESEYLRQVVEPWFTVCESKVPRTLTDVVHRIL
RKGDGKAHRMLRGTPNGDDHSVYTKFGSGISPAPSRLVVLSESLQEVQRPQLYRVSRLR
SSSTPHRVVLCEVMSAFSPLNVEEMECTGVLGKLSDTSSVTLNVVDPGYVLTMIRVCSC
SAMRVKRWTWTSFFNVKERLEDRWRIPPDPKECESHCEPIFAGEVTSAVRGSVPDYCYA

WMSTCEVQGDVYQVSLGTAKFHRFLNQIRAPFIVDDPCTPSAPCKGSGDSLVLAKIEED
NPRFTTMNGELTPRYNWDTHKVYQVHLPGVTTSILDASCGFLHGGYVYYQLMSGRIVS
VSVGTLQFGDKVKPPICTEWKGPYMPFVLPDSQVASTSEQLRQDLLHCQTRKEVVLNA
LATKRLPSITLFEGLGYKGSESYGLVSRKGLLYAAPCPSVEYTDLEHVEGNIWMVVNNG
REVGCLDGGLNFAVKSGCVVNPNASVSILLGEWKVISDRDGKLLAEPIPKAGWGSIPAL
ENISAAFGDYLASLEQPPLWDDGNGPIIIPTSNSTGDPVIHSGASSLWSSMSLASKITAILM
PLLSLAVVVGIIMCCRR.

U1 Gene RFFRV

MSCLTQDLRKEIRERVSNSISQNPSVTTLIIIEVILWVGTLAYISIALGCHRYLQARIKNSVE
RRQRFRGDGVLLPDTGPA.

U2 Gene RFFRV

MEEKCSDSDYFKELTDAAIEGVWASPLYPITLATVIIFLILLIFVVAWRAAVIAKIRHRIDE
STARRLRPDFGGRP.

U3 Gene RFFRV

MSPQHGASALISGVDPDHFSLIIESYPILVGNYAATLILTVTLVLSFSLATIIFFFSSVNNLT
DIRGVLIYHLGNEVSEFASHALAAVCSAQSTSARLTRELEQFRLSGQVTEVTPSSGATGE
LSIPQRYRMLLVEKDMLENEMFIAEHSLV.

L Gene RFFRV

MEKSFLFDRIPDSVLHSPLILREELCFCRPDKETSDYPAIPQPDEFTISRRPELIELRSLSLTG
AISGVGCIKWLAYMRLNWIEAKDIMRLMAEEYQSSTIHPEIASDFNNVCEGLFSRLHDQ
WGENQHPSALQDIWNVFVAQYQSDAIIDNWTKYRILSAALLWSNHKERGNQSEFQNSII
AKAGWGVRAINGVEVITHKLLTKRERLGKLTVGAYWCLLELANQPPRLFRKSLLLCEK
DLYLQRWISLFLCNCQNAFGGIPLPQPKHLIKMYRMGDIILATEGNEGYNAIKTIESIMVG
SAHKQLEDESISPTGDKFYEFITKXGLSACHLESSSSAMRKIIHIGEIVTPEVAIEMAGCYR
HWGHPIINPVGGLTAVRENATAQLPTNERLMISLAADLNYLLLRSYFEEHGRWPPGVRY
EAAPEIAKDLFNKWVSTNQFPGPTSASQVRNSWFFVTYDSLFDKNQEIPILSLISDKSHSV
GRSALSTMCLKKNLLLSPSRRVLQSTLSYAEIDVNKFLDSIDSTENGLSNDDLVILLREKE
RELKVKGRFFSLMTYKLRTYFTATEYLIAKHILPVLPEITMMQGQIDLWKTFKGAVRTV
SQEKSTHHMIHVDFEKWNNFQREESTAPVFQIMDRAFGWSNVISRTHNFFSRCFVGYAG
RIDMFPIGLTDNWPWCWTGHKGGFEGLRQKGWSVVGALLIRHVMRLTGLHGKVLIQG

DNQVIILEYPLASSSNTSSLALERHRHSKMTTHFLSVFSELSKSIGLRIKPEETWISSRMVY
YGKFPVIDGSARGMVLKKLCRIFAISNDLTPSISNSISSLHTSCIAGCIQSSYAVPIYMFAIF
YGFFLLHDFFEYNPLSMEPMIFTFRRALFGTTAVRDITKVRAPTTKDSIIISVARDWAPAS
NKQQSALGLDLLTRDSSLGGLGGASILKYMIRQFPDPVTEGLSFAKVGLSKAETPELKTI
FSSYGGLPIKSGSVELLLEAPMSINLPSSTRPVNVLRRFVEEKITQNMDTLVNNQLVYQA
LSMKQDMRPPFLKMCASLQPFFPRFASSLYSATPIGVAETIVGKFLGTKTVARIALREGA
GQLGKKLVESEVELITTYLTVLKLSALEHWGCSSKLADSYRSKSWGGQIWGVTVPHPAE
QFYLEWPMTSGCFKESLAWMNDFAEETVKVSLNERMIYGDTSPRSAGPMNPYLGGRTS
ERSSVQSWLELETSSSIAKRLVELINACGWAYQKGSHLHHAIKEMLTQLTGLVIPSDAVC
RITESGSLGHRFSDPRVSSGAMSATNYNTATHIAFSTNDLVRLKRGEDNYMVLFQGLFV
YFTSIIGELVRRGDNGSPTAHLHPKCEECMIPCPDVSMELSNPVVPLYSMFLSPRLPNDFL
TLPENLYVDIDPFKVKPDQIKTIVNIVPAYSTVSVDTLSGEEISDAISNVAAIIAVTKTNPQ
GRWTLGIRTAIPVYAGYKCSWKVLKSKIVFWTFAMLAFRHINESEEHDCCEACQHILRK
QAADHWNLHNPTMLGGMLSNTDIIVELAKEQIALSAGFPISPMELSSAAGQSLFEQCVS
HGKEFSLPEFPKGVIALPRDMTGGGTPAYLIACMMCYAGFFKGANTGPLKALAGLTTE
MKTSVIASQAFEESEGVWSRLVDMLHENTSTLCCVLDIDFKTLTKRGQDVDHILPGSIPA
ISLDTVRGAYCIWNTASTTKGPESSYLEEEGEPEVTQTPIMHLFRPILMSANGSIKILSVLK
TIQLQPKNVAVVGDGNGGFLRALLGLYPTAEFFFNSLSEMSNYGDQGEFTNYPACVLDS
PSDWGRVINLGEVLTLPSDLSSERWPSEVTKYWMKKCFDPDLLVCDAESYDSIKRSEILS
NLLKVCGQRSHVIIKAHLSRMQDVQSLVNAHLHSHNVMYLRAPYSNWGKFEVYLWYT
RIVERNDMRYAPKDTYLMWTRAEFNAHPQLQLTAFHSWVFPHLEKDDRLRAVRRMLS
LRRHISRYTKIRPQELSRVERKAKEHGDMGFCELLLHYLGSKFISSMNNSVIRATSHAGR
LRASLKRSELIGLIGTAIGASCLVSAISHPHDYCLFLELYKNGVDVVLGNDKGNIKPIVVR
AWSGESTPVTITPKELKKPVHSMVRYLGGLLWSSGIDRITQRLEWLVREKYELGFSEGY
NHLAVATWDNDTLDVDPLECIRWEMEQAAVEEIDFGGIIDE.
